# Valuable Genomes: Taxonomy and Archetypes of Business Models in Direct-to-Consumer Genetic Testing

**DOI:** 10.2196/14890

**Published:** 2020-01-21

**Authors:** Scott Thiebes, Philipp A Toussaint, Jaehyeon Ju, Jae-Hyeon Ahn, Kalle Lyytinen, Ali Sunyaev

**Affiliations:** 1 Department of Economics and Management Karlsruhe Institute of Technology Karlsruhe Germany; 2 Desautels Faculty of Management McGill University Montreal, QC Canada; 3 College of Business Korea Advanced Institute of Science and Technology Seoul Republic of Korea; 4 Department of Design and Innovation Case Western Reserve University Cleveland, OH United States

**Keywords:** genomics, genetic testing, genetic privacy, direct-to-consumer screening and testing, taxonomy, cluster analysis

## Abstract

**Background:**

Recent progress in genome data collection and analysis technologies has led to a surge of direct-to-consumer (DTC) genetic testing services. Owing to the clinical value and sensitivity of genomic data, as well as uncertainty and hearsay surrounding business practices of DTC genetic testing service providers, DTC genetic testing has faced significant criticism by researchers and practitioners. Research in this area has centered on ethical and legal implications of providing genetic tests directly to consumers, but we still lack a more profound understanding of how businesses in the DTC genetic testing markets work and provide value to different stakeholders.

**Objective:**

The aim of this study was to address the lack of knowledge concerning business models of DTC genetic testing services by systematically identifying the salient properties of various DTC genetic testing service business models as well as discerning dominant business models in the market.

**Methods:**

We employed a 3-phased research approach. In phase 1, we set up a database of 277 DTC genetic testing services. In phase 2, we drew on these data as well as conceptual models of DTC genetic testing services and iteratively developed a taxonomy of DTC genetic testing service business models. In phase 3, we used a 2-stage clustering method to cluster the 277 services that we identified during phase 1 and derived 6 dominant archetypes of DTC genetic testing service business models.

**Results:**

The contributions of this research are 2-fold. First, we provided a first of its kind, systematically developed taxonomy of DTC genetic testing service business models consisting of 15 dimensions in 4 categories. Each dimension comprises 2 to 5 characteristics and captures relevant aspects of DTC genetic testing service business models. Second, we derived 6 archetypes of DTC genetic testing service business models named as follows: (1) low-cost DTC genomics for enthusiasts, (2) high-privacy DTC genomics for enthusiasts, (3) specific information tests, (4) simple health tests, (5) basic low-value DTC genomics, and (6) comprehensive tests and low data processing.

**Conclusions:**

Our analysis paints a much more complex business landscape in the DTC genetic testing market than previously anticipated. This calls for further research on business models and their effects that underlie DTC genetic testing services and invites specific regulatory interventions to protect consumers and level the playing field.

## Introduction

### Background and Objectives

When 23andMe opened its Web store to the public in 2007, it was among the first in the second wave of the so-called direct-to-consumer (DTC) genetic testing services that publicized the idea of personal genomics [1]. The surge of DTC genetic testing services such as 23andMe, Ancestry.com, or FamilyTreeDNA, to name a few, during the last decade is thereby mainly driven by the rapidly declining costs for collecting and analyzing genome data [2] and the public’s growing interest in genomics [3]. Overall, estimates suggest that the global DTC genetic testing market will be worth approximately US $610 million by 2026 [4].

DTC genetic testing refers to genetic tests targeted toward consumers that do not require the involvement of a medical professional in mediating the service and interpreting it [5]. Typical tests offered by DTC genetic testing services include ancestry tests, nonmedical lifestyle tests (eg, traits, fitness, nutrition), and medical tests (eg, carrier status, genetic health) [3]. Compared with conventional medical testing, DTC genetic tests are, however, initiated by consumers, not their physicians, and sold directly to consumers over the internet [6]. Consequently, consumers are responsible for controlling and managing their genetic information on the Web, as well as choosing an interpreter for their genetic information or interpreting it themselves [6]. Despite the ongoing proliferation of genetic testing and the public’s rising interest therein, DTC genetic testing has been a source of controversy ever since the inauguration of the first services offering such tests [5]. This especially pertains to business practices underlying DTC genetic testing services, as it is often believed that such services resell access to their consumers’ genetic data to increase revenue and to compensate for selling budget-priced tests. 23andMe, for example, uses its DTC front end to build a health information database and sells access to this database to clinical research and biopharmaceutical companies [7]. Concerns about the impact and business practices of DTC genetic testing services climaxed in several professional associations recommending to consumers to refrain from using DTC genetic testing services at all [5] and the US Food and Drug Administration (FDA) sending out cease and desist letters to several DTC genetic testing services in 2013 [6].

To realize the full potential of a disruptive technology such as genetic testing, it needs to be met by innovative business models [8]. Toward this end, DTC genetic testing can be regarded as a class of business models that can serve to make genetic testing more affordable and accessible. In light of the glaring contradiction between the ongoing proliferation of DTC genetic testing services, on the one hand, and the spreading skepticism toward their business practices on the other hand [9], however, there still seems to be little consensus among providers of such services, policy makers, professionals, and the general public on what actually constitutes suitable business models for DTC genetic testing. Several DTC genetic testing services, for example, recently started to shift their business models toward greater involvement of medical professionals and test approvals by regulatory bodies [6]. This resulted in the FDA-granting approvals for certain DTC genetic tests [6], which is likely to provide additional impetus to this market. Thus far, research provides little guidance for assessing or developing business models in the context of DTC genetic testing. Owing to the clinical value and sensitivity of genome data, past research on DTC genetic testing has primarily focused on ethical and legal issues surrounding genetic tests offered directly to consumers [10], including impact and clinical utility of DTC genetic tests [11], as well as studies of awareness and perceptions of DTC genetics and its risks [12-14]. Most of our knowledge about business models of DTC genetic testing services today stems from newspaper articles [15], blogposts discussing specific DTC genetic testing services [7], or company reports [16]. To the best of our knowledge, so far only a single white paper explicitly investigates business models underlying DTC genetic testing services (cf, the Business Models in Genomics section), and there are no published academic articles on this topic. A first step toward closing this knowledge gap pertains to scrutinizing the status quo and understanding what characterizes business models in the DTC genetic testing context today as well as what, if any, dominant business models have emerged in this market. We therefore ask the following research questions:

- RQ1: What are salient properties of DTC genetic testing service business models?
- RQ2: What dominant business models of DTC genetic testing services have emerged?

To answer these research questions, we develop a taxonomy of DTC genetic testing service business models (RQ1) and subsequently draw on this taxonomy to derive archetypes of DTC genetic testing service business models (RQ2). In doing so, we provide a systematic classification of DTC genetic testing service business models and expose their essential characteristics. We posit that the knowledge encapsulated in the taxonomy and dominant archetypes makes fruitful contributions to research on the role and impact of building innovative (internet-based) business models in health care. It can aid policy makers to better understand how DTC genetic testing services operate and design policies and regulations to protect consumers and enable fair competition when possible while realizing their full potential. Moreover, it should also help addressing health professionals’ concerns regarding the impact of these services and increase consumers’ awareness to make more informed decisions about using such services. Finally, for nascent businesses in the DTC genetic testing industry, our findings can serve as a blueprint of the emerging competitive landscape and as a roadmap to advance their business models.

### Business Models in Genomics

Despite the term business model being commonly used in management and strategy, little consensus prevails over what constitutes a business model [17]. Extant literature has proposed an abundance of definitions for business models [8,18,19]. Drawing on Shafer et al [17], who synthesized extant conceptualizations of business models within scientific literature, we understand a business model as “a representation of a firm’s underlying core logic and strategic choices for creating and capturing value within a value network.” Consequently, business models comprise 4 major categories of components: (1) *strategic choices* (eg, customers, target markets, value propositions, revenues and pricing, competitors), (2) *value creation* (eg, key resources, assets, processes), (3) *value network* (eg, information and product flows between an organization, its suppliers, and customers), and (4) *capturing value* (eg, profit-making mechanisms).

As pointed out earlier, disruptive technologies like genetic testing must be met by innovative business models to realize their full potential [8]. However, the health care sector is knowingly slow in adopting disruptive technologies, especially the innovative business models that these technologies afford [8,20]. Looking at genetic testing, we see that this nascent but arguably increasingly important aspect of modern health care is no exception to this phenomenon. Toward this end, scientific literature on the business aspects of DTC genetic testing services, especially their underlying business models, remains scarce [21]. The stream of research that is closest to this topic relates to socioeconomic research on DTC genetic testing [1]. Research in this stream is concerned with the marketing strategies of DTC genetic testing services and their impact on consumers [22,23], as well as economic implications of consumers freely sharing their genome data [24-26]. Extant literature has also covered the history [27] and size [28] of the global DTC genetic testing market. Our review of the related literature resulted, however, in only 1 white paper by Vanhala and Reijonsaari [29] that explicitly investigates business models in the context of DTC genetic testing. They define a business model as connecting consumers’ needs with the solution offered by a service. Accordingly, any DTC genetic testing service business model comprises value propositions, distribution channels, revenue logic, customer segments, and key resources (eg, genome data). On the basis of these 5 business model aspects, Vanhala and Reijonsaari [29] further derive 5 categories of DTC genetic testing service business models: (1) comprehensive genomic tests for consumers and as genome data bank material (eg, 23andMe), (2) genomics as part of individual health planning (eg, MD Revolution), (3) genomic services based on comprehensive genetic testing (eg, Genetrainer), (4) medical precision tests for consumers (eg, Myriad Genetics), and (5) restricted trait tests (eg, Genecodebook Oy) [29]. Although this categorization certainly is a valuable step toward shedding light onto DTC genetic testing service business models, the authors provide little information on the employed methodology and base their analysis on assessing only a limited number of DTC genetic testing services. Adding to this, the study was published in 2013 and thus does not account for recent changes in the landscape of DTC genetic testing service business models.

## Methods

### Overview

To answer our research questions, we adopted the 3-phased approach of Remane et al [30]. A detailed description of each phase is given later, and Table 1 provides a brief summary of the individual phases.

**Table 1:** Overview of the research approach.

Phases	Phase 1: Database setup	Phase 2: Taxonomy development	Phase 3: Cluster analysis
Inputs	Desk research Web-based genetic testing service repositories [31-33]	DTC^a^ genomics literature (deductive iterations) List of DTC genetic testing services (inductive iterations)	DTC genetic testing service business model taxonomy
Steps	Compile a list of DTC genetic testing services Filter services not available anymore Collect information about services from multiple sources (eg, websites, blogs, research, or news articles)	Define a meta-characteristic Develop taxonomy iteratively until all ending conditions are met	Identify suitable numbers of clusters (Ward’s method) Run iterative partitioning algorithm with the identified numbers of suitable clusters Select the most fit cluster solution Analyze cluster solution and derive archetypes
Outcomes	List of 277 DTC genetic testing services	Taxonomy of DTC genetic testing services' business models with 41 characteristics in 15 dimensions	6 archetypes of DTC genetic testing service business models

^a^DTC: direct-to-consumer.

### Phase 1: Database Setup

In phase 1, a database of DTC genetic testing services was set up. It served as a basis for taxonomy development and cluster analysis in phases 2 and 3, respectively. As we are solely interested in DTC genetic testing services that have a Web presence, the internet provided a good starting point to collect information about these kinds of services. We therefore conducted a desk search using the internet to set up our database. Our search led to the identification of 3 central resources that list a large body of genomic service providers (not limited to DTC genetic testing services). First, Phillips [31] has compiled a comprehensive list of 301 DTC genetic testing services, which is available on her website. Second, DNA Testing Choice [32], a UK-based news and reviews service of genetic tests, offers rankings for a variety of DTC genetic tests. As some DTC genetic testing services may offer more than one test, they may also appear in multiple rankings on DNA Testing Choice. Finally, the International Society of Genetic Genealogy [33] also provides a list of DTC genetic testing services. Overall, all 3 sources together listed 428 genetic testing services. For each service, we noted its name, website, a brief service description, and relevant sources (ie, websites that reference the respective service). As the focus of this study is on DTC genetic testing services, we screened all entries for DTC genetic tests before the taxonomy development process. This led to the exclusion of 171 services that were either not available anymore or did not fit our definition of DTC genetic testing services (eg, services that offered genetic testing of animals). We further identified 20 additional DTC genetic testing services while collecting information about services already included. In total, our final database contains 277 DTC genetic testing services. A complete list of services included in and excluded from our database can be found in Multimedia Appendix 1.

### Phase 2: Taxonomy Development

The second phase focused on the development of a taxonomy of DTC genetic testing service business models (thus answering RQ1). Taxonomies are important tools in scientific disciplines as they provide researchers with fundamental categories to analyze and understand complex domains [34]. We based the development of our taxonomy on the method by Nickerson et al [34]. It provides a systematic approach (as opposed to an ad hoc approach) to taxonomy development that combines inductive and deductive reasoning and has been extensively used to systematically develop taxonomies for phenomena in health care [35-42] and other domains [30,34,43,44]. The method consists of 7 iterative steps and provides guidelines for each step in the taxonomy development process. Step 1 abides the selection of a so-called meta-characteristic. Being the most comprehensive characteristic, the meta-characteristic serves to avoid a situation of naïve empiricism, acting as the basis for the choice of characteristics included in the taxonomy [34]. Each characteristic in the taxonomy must therefore be a logical consequence of the meta-characteristic [34]. Drawing on RQ1, we defined our meta-characteristic as *salient properties of business models of DTC genetic testing services*. As steps 3 to 7 of the taxonomy development are iterative, some predetermined conditions that end the process must be defined in step 2. For this research, we adopted the 5 subjective ending conditions (ie, conciseness, robustness, comprehensiveness, extendibility, and explanatory) and 8 objective ending conditions (eg, characteristics in a dimension are mutually exclusive, and there were no changes made to the taxonomy during the last iteration) provided by Nickerson et al [34]. Next, steps 3 to 7 involve the iterative development of the taxonomy (cf, Multimedia Appendix 2), whereby each iteration starts by choosing either a conceptual-to-empirical (deductive) or empirical-to-conceptual (inductive) approach in step 3 and ends with a review of whether the ending conditions are met in step 7 [34]. If the ending conditions are not met, an additional iteration is performed.

**Figure 1:**
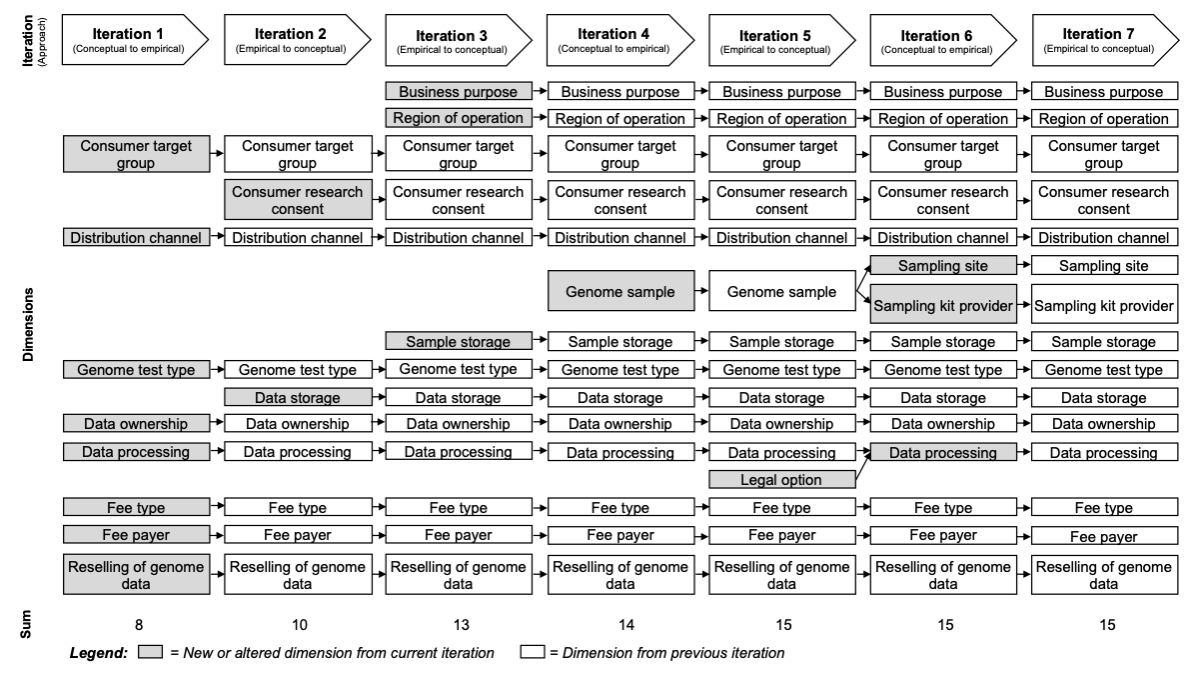
Overview of the taxonomy development iterations.

In total, we performed 7 iterations. An overview of the development of dimensions for individual iterations is given in Figure 1. The first iteration followed the conceptual-to-empirical approach and was based on the model of Vanhala and Reijonsaari [29]. It led to an initial taxonomy with 8 dimensions. We classified DTC genetic testing services examined by Vanhala and Reijonsaari [29] and the top 10 ancestry, paternity, and health test service providers according to DNA Testing Choice [32] in the second iteration to verify the validity of our initial taxonomy. This led to the addition of 2 new dimensions.

Before the next iteration, we grouped all services based on the tests they offered. Similar to DNA Testing Choice’s ranking categories, we grouped services into 1 of the 3 categories: (1) *genomics enthusiasts* (eg, ancestry, genetic dating, trait testing), (2) *relationship tests* (eg, paternity, maternity), and (3) *health tests*. Like iteration 2, iteration 3 also followed the empirical-to-conceptual approach. After the analysis of the *genomics enthusiasts* subset, we added 3 dimensions. At this point, a review of dimensions and characteristics in iteration 4 (conceptual-to-empirical) resulted in the addition of 1 further dimension. In iteration 5 (empirical-to-conceptual), we analyzed all services offering *relationship tests*, which led to the addition of 1 new dimension. The following iteration 6 again followed the conceptual-to-empirical approach. Within this iteration, we merged 2 dimensions and split 1 dimension into 2 distinct dimensions. During iteration 7, the remaining subset of *Health Tests* was examined, which led to no alterations to the taxonomy. At this stage, the taxonomy fulfilled all ending conditions after 7 iterations (cf, Multimedia Appendix 3).

### Phase 3: Cluster Analysis

The third phase focused on deriving archetypes of DTC genetic testing service business models (RQ2) using the previously created taxonomy as a baseline for cluster analysis. Cluster analysis is a process of finding distinct groups of objects (ie, clusters) in data [45]. The objective is to find groups (clusters) for which the objects of 1 group are highly similar in selected attributes, whereas they are as dissimilar as possible from objects in the other groups [45]. An abundance of clustering methods is available, and choosing the most suitable method can be cumbersome and error prone in terms of what similarity or dissimilarity measure to choose, how many clusters to generate, or overall performance of clustering algorithms [46]. Iterative partitioning algorithms such as *k*-means, for example, provide better performance than hierarchical clustering methods, but in turn usually require the a priori definition of how many clusters to produce [46]. To address these problems, we adopted the 2-stage clustering approach suggested by Punj and Stewart [46]. In this process, the first stage utilizes a hierarchical method to determine a preliminary solution, which can be used to deduce candidate numbers of clusters that serve as starting points for the iterative partitioning algorithm in stage 2. On the basis of the candidate number of clusters obtained in stage 1, an iterative partitioning algorithm arranges the included objects in their final cluster solution in stage 2 [46]. According to Remane et al [30], we utilized Ward’s method for stage 1 and the *k*-means algorithm for stage 2.

Ward’s method is an agglomerative clustering procedure. It starts by combining the 2 objects closest to each other into 1 cluster and repeats this process until all objects belong to the same cluster [47]. During each iteration, the similarity between 2 clusters is calculated by the number of identical characteristics. As our taxonomy holds only binary data (ie, a characteristic is either applicable or not), the squared Euclidean distance, which places progressively greater weight on objects that are further apart from each other, is a suitable similarity measure [30]. The dendrogram produced by Ward’s method indicated that a 4-, 5-, or 6-cluster solution would have the most explanatory power for our dataset. Also, by reviewing the scree plot, the elbow rule suggested the 4-, 5-, 7-, or 9-cluster solution in this particular order. With the preliminary cluster solutions in place, we used the *k*-means method to derive our final cluster solution in the second stage. The *k*-means method produces a partition of the dataset into an a priori defined number of clusters [47]. Starting with an initial partition, objects are moved into other clusters if they are closer to its mean vector than that of their current cluster, usually calculated using Euclidean distance. After each iteration, the mean vectors are updated [47]. The procedure continues until all objects are closer to the mean vector of their own cluster than to the mean vectors of any of the other clusters or no significant changes are found [47]. For the 4, 5, 6, 7, and 9 cluster solutions, the algorithm ran through 10, 17, 9, 13, and 12 iterations before achieving convergence, respectively. However, retrieved significance values for characteristics (ie, how relevant a certain characteristic is for the cluster solution) indicated that the 4 and 5 cluster solutions were of inadequate quality because they possessed too many irrelevant characteristics (cf, Multimedia Appendix 4). We therefore did not consider those cluster solutions any further. The remaining cluster solutions (ie, 6, 7, and 9 clusters) were next manually compared for their explanatory power by 2 researchers (ie, we sought to find meaningful interpretations for all clusters in all cluster solutions). Although we were able to find meaningful interpretations for the 7-cluster solution, it produced entirely different clusters, which we deemed less meaningful and providing little to no additional insight compared with the 6-cluster solution. For the 9-cluster solution, on the other hand, we were unable to find meaningful interpretations for all clusters. Hence, we selected the 6-cluster solution as the most suitable one for this study and report it below.

## Results

### Direct-to-Consumer Genetic Testing Service Business Models Taxonomy

Our final taxonomy consists of 15 dimensions. Each dimension consists of 2 to 4 characteristics, with a total of 41 characteristics. Furthermore, the dimensions have been grouped into 4 categories based on the previously outlined categories of components of business models (ie, strategic choices, value network, create value, and capture value) to provide a better understanding of how the dimensions relate to each other. Table 2 offers an overview of the final taxonomy, whereas the results of our coding can be found in Multimedia Appendix 5. In the following, we describe each dimension and its characteristics in detail.

**Table 2:** Taxonomy of direct-to-consumer genetic testing services’ business models.

Dimension		Characteristics
**Strategic choices**		
	Business purpose	For profit; nonprofit
	Region of operation	Local; worldwide
	Consumer target group	Enthusiasts; specific information seekers; enthusiasts and specific information seekers; chronic health issue and risk group
	Consumer research consent	Mandatory; optional; data not used
**Value network**		
	Distribution channel	Internet only; health care professionals only; multicontact service
	Sampling site	Home collection; lab collection; home and lab collection
	Sampling kit provider	Service provider; third party; service provider and third party
	Sample storage	Never; mandatory; consumer decision
**Create value**		
	Genome test type	Genotyping; sequencing; genotyping and sequencing
	Data storage	No storage; isolated storage; database for service provider
	Data ownership	Consumer; service provider
	Data processing	No interpretation; basic interpretation; value-added interpretation
**Capture value**		
	Fee type	Pay-per-use; pay-per-use and subscription; no fee
	Fee payer	Consumer only; consumer and health insurance
	Reselling of genome data	Yes; no

#### Strategic Choices

The first category of components of a business model entails 4 dimensions related to strategic choices of a DTC genetic testing service, such as target markets and customers. The *business purpose* dimension answers the basic question of whether a service provider seeks to generate profit or whether it is a nonprofit organization that contributes to genomics research and makes DTC genetic testing more accessible to consumers. The *region of operation* dimension answers the question where a DTC genetic testing service is offered. In our taxonomy, we distinguish between the characteristics local (ie, tests are only available in the country the service has been registered in) and worldwide (ie, tests are offered all over the world with the exception of countries that do not allow such tests by law). The third dimension, *consumer target group*, divides DTC genetic testing services into 4 target groups. Products aimed at enthusiasts seek to spark the curiosity of the consumer for information on their DNA such as ancestry, taste tests, lactose intolerance, or health traits [29]. The second target group concerns specific information seekers. Services for this target group offer genetic tests that aim to answer a specific question and include, for example, paternity and other relationship tests, immigration tests, or tests for certain diseases. There are also genetic testing services that target both enthusiasts and specific information seekers and are summarized in the third characteristic of this dimension. The last target group addresses consumers dealing with chronic health issues and related risks (eg, individuals with diabetes, individuals with high blood pressure, or individuals with an increased risk of cancer). Finally, the *consumer research consent option* dimension indicates whether DTC genetic testing service providers ask for consumers’ consent to use their data for research. For some services, it is mandatory that consumers give their consent for using their data for research to purchase the service. Other service providers either give consumers the option to consent into their personal genome data being used for research or do not utilize their customers’ data for research purposes at all.

#### Value Network

The value network category comprises 4 dimensions that characterize a DTC genetic testing service’s relationship to key partners and customers, as well as how information, products, and services flow through this network. The *distribution channel* dimension describes how products and services are communicated and offered to the consumer. Although many DTC genetic testing service providers only offer an internet presence for all consumer means, some service providers may require a health care professional to be involved. However, health care professionals mainly serve as a means for distributing and, in some cases, carrying out sample collection and follow-up patient counseling but are responsible neither for performing the actual genetic testing nor for interpreting test results. The third and most comprehensive distribution channel is described as a multicontact service [29]. This includes internet solutions, mobile apps, telephone consulting, stores, and home visits to offer the product to the consumers. The s*ampling site* dimension summarizes where a consumer’s genome sample is collected. Many services offer the collection of genetic material via home collection kits that are mailed to their consumers. The sample is then taken by the consumers themselves (eg, buccal swab or saliva sample) and sent back for analysis. Other services require their consumers to visit a lab, where samples are taken by the staff. Lab collection is usually offered either as a convenience to the consumer or because it is legally required (eg, a paternity test that is to be acknowledged by the court). Some services offer home and lab collection of genome samples. Adding to this, the *sampling kit provider* dimension describes whether a service provider offers their own sample collection kit or whether a third-party sample collection kit is used. Some services offer both, their own sample collection kit and the option to use a third-party kit. Finally, the *sample storage* dimension indicates whether the collected sample is destroyed (ie, never stored) or kept after the genome data have been generated. Although for some genetic testing services it is mandatory that genetic samples are kept (eg, for legally binding paternity tests), others leave this decision up to their customers.

#### Create Value

The third category contains 4 dimensions, which describe how DTC genetic testing services create value for customers. It thus focuses on their products and processes. The *genome test type* dimension determines what kind of method is used to generate genome data and consists of three characteristics. A service provider may either offer genotyping only, sequencing (whole genome or whole exome) only, or both, genotyping and sequencing. Next, the *data storage* dimension considers how DTC genetic testing services store the genome data of their consumers. Service providers can either not store the produced data, store it isolated for the consumers’ access only, or store it in a common database, which is used to improve service quality. If the data are not stored, then they are deleted shortly after the consumers retrieve their genome data. Some services collect fees for the isolated storage of the data and will only keep it as long as the consumers decide to store it. The *data ownership* dimension classifies DTC genetic testing services in terms of who owns the genome data. By purchasing a product from a service, the consumers agree to their terms of service. The terms of service usually state whether the collected genome data are the property of the consumers or the service provider. If the ownership stays with the consumers, the decision power over the data also remains with the consumers. If a business claims ownership of the data, then it is authorized to use the data without restrictions to further the company’s interests. Finally, the fourth and last dimension, *data processing*, describes the degree of genome data analysis provided by a service. Some DTC genetic testing services do not offer any interpretation but instead deliver the raw genome data by means of genotyping or DNA sequencing, only. If services offer interpretation of the produced genome data, they usually create reports on certain information. Most services provide an analysis of the genome data in terms of ancestry information, health traits, paternity tests, or cancer tests. Alternatively, some services offer value-added interpretation, whereby they augment their interpretation with additional services such as, for example, carrying out legally binding paternity tests (as opposed to cheaper nonlegally binding tests) or providing diet plans or supplements based on the analysis of their consumers’ genome.

#### Capture Value

Capture value is the final category of business model components. It pertains to three dimensions that describe how DTC genetic testing service providers generate revenue. The *fee-type* dimension is concerned with the providers’ pricing model. Consumers may be charged on a per-use basis or on a per-use basis paired with a subscription model (eg, to account for additional services besides the actual test). Some tests are offered to consumers free of charge. The *fee-payer* dimension describes who pays for the offered tests. Tests can be paid for entirely by consumers themselves as well as partly or completely by the consumers’ health insurances. The *reselling of genome data* dimension relates to whether a service provider generates revenue from reselling collected genome data to third parties. Possible customers for genome data include research institutes, clinics, or pharmaceutical companies.

### Archetypes of Direct-to-Consumer Genetic Testing Services’ Business Models

The clusters in the 6-cluster solution comprise between 21 and 73 of the 277 DTC genomics services in our database. Thereby, each cluster has a different focus regarding the dimensions and characteristics of the DTC genetic testing service business model taxonomy. As the taxonomy development method by Nickerson et al [34] results in characteristics that are mutually exclusive and collectively exhaustive, the data can be interpreted as percentages. For example, 89% (42/47) of the companies in cluster 1 operate worldwide whereas 11% (5/47) offer services in their respective country only. Table MA6-1 in Multimedia Appendix 6 provides an overview of the results of the cluster analysis, whereby darker colors represent higher percentages of services in the cluster belonging to a characteristic for the corresponding dimension. We elaborate on each cluster below by highlighting its most representative characteristics and providing examples of typical DTC genomics services.

#### Cluster 1: Low-Cost Direct-to-Consumer Genomics for Enthusiasts

**Figure 2:**
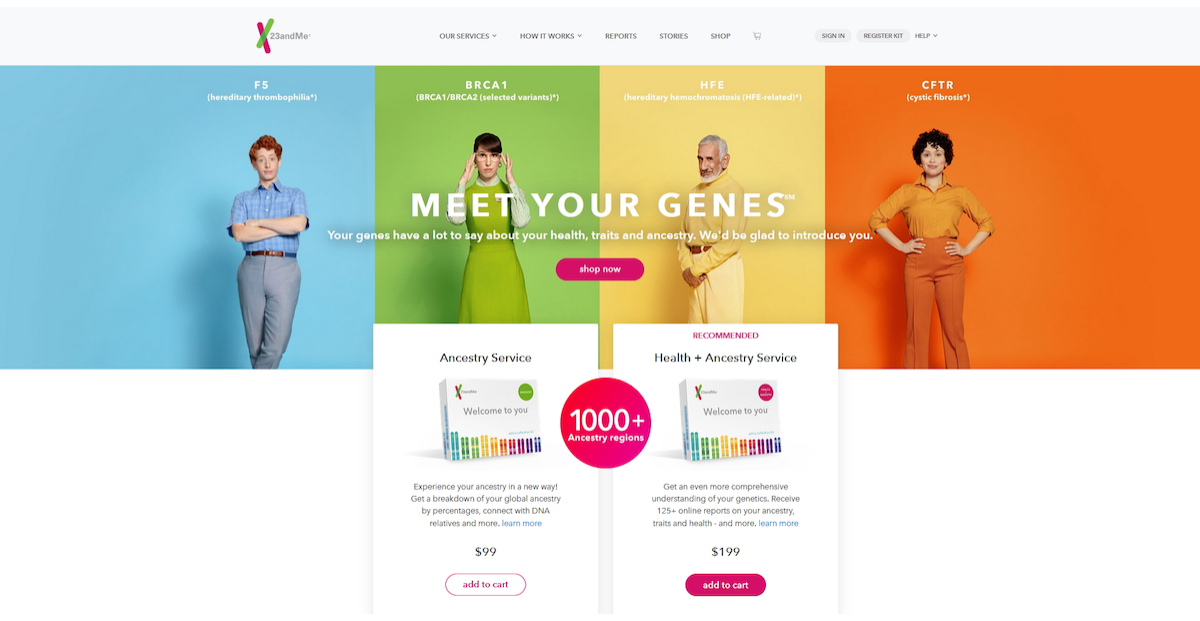
Screenshot of the 23andMe website from October 2019.

The first cluster combines the low-cost genotyping tests offered by companies like 23andMe (see Figure 2 for a screenshot of their website) with the costlier sequencing tests provided by companies like Veritas Genetics who strive to make sequencing affordable for the enthusiast DTC market. These companies operate worldwide as they provide their services over the internet and less via multicontact channels. Most operators like 23andMe only charge the consumers a 1-time fee for their home collection kit but claim the rights of the produced genome data and utilize it to improve their own services and resell it for profit to compensate for the relatively low prices of their tests. Other services like Ancestry.com operate on a pay-per-use and subscription fee model and therefore leave the ownership of the data with the consumer. Finally, some services offer health-related tests that are eligible for insurance coverage. Although the offered services are value-added tests, services either make it mandatory or give customers the option to willingly participate in research with their data. Other prominent examples of services in this cluster are FamilyTreeDNA, MyHeritageDNA, or the Genographic Project.

#### Cluster 2: High-Privacy Direct-to-Consumer Genomics for Enthusiasts

Cluster 2 is in many aspects similar to cluster 1 but differentiates itself in the crucial dimensions concerning the customers’ data privacy. Most services in cluster 2, such as African Ancestry, provide internet only or internet and telephone solutions for genomic enthusiasts. African Ancestry provides a simple genotyping test that answers the general question of whether a customer has origins tracing to Africa and other information regarding their African ancestry. The tests can only be taken with a home collection kit and results are made available on the Web. The generated genome data remain the property of the consumer for all services in this cluster, and the genome data are either erased or stored in an isolated storage accessible by the consumer only. It is not sold for revenue. Furthermore, the data are usually not used for research, but consumers may be given the option to provide their data. The costs for these tests are covered by a 1-time fee for the consumer or an additional subscription. Other examples of services in this cluster include EasyDNA, FitGenes, or The Makings of Me.

#### Cluster 3: Specific Information Tests

**Figure 3:**
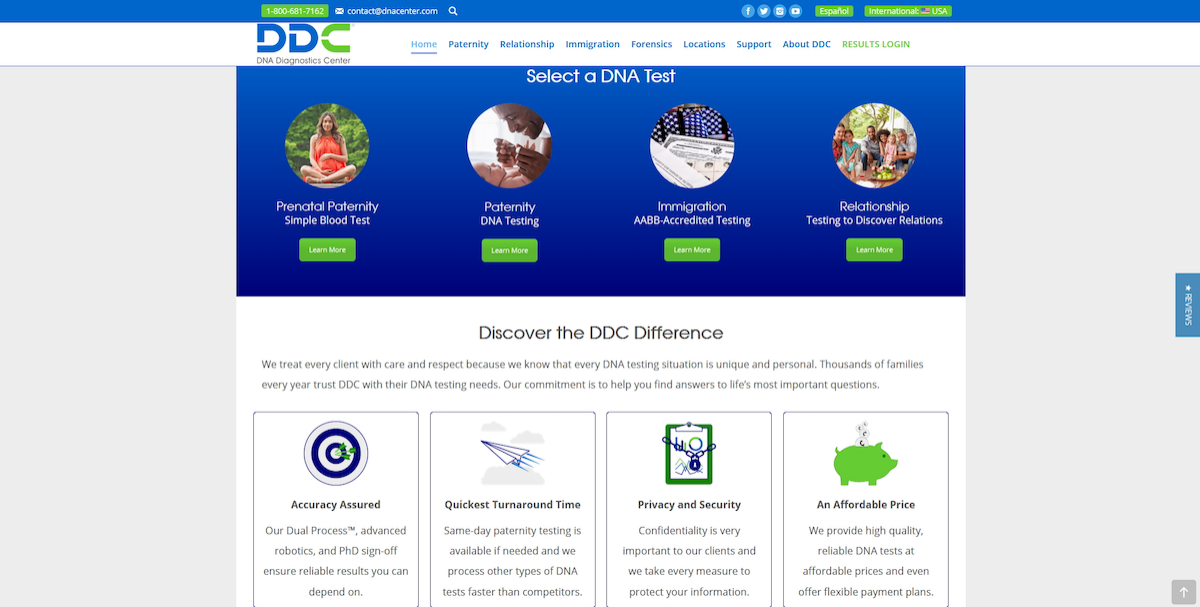
Screenshot of the DNA Diagnostics Center website from October 2019.

The third cluster holds DTC genetic testing services that provide consumers with solutions to specific questions and aim to provide a more elaborate service with value-added data processing. One prominent example for this group is DNA Diagnostics Center (see Figure 3 for a screenshot of their website). Their choice of distribution channel over the internet and via telephone allows for a worldwide service. DNA Diagnostics Center offers a variety of genome tests on specific information ranging from relationship to forensics on a 1-time cost basis for the customer. Although all genome tests of this cluster cover genotyping, the sample collection can either be performed by the consumers themselves with a service-provided home collection kit or by a professional (eg, for a legal paternity test at a laboratory operated by DNA Diagnostics Center). The genome data are primarily stored in isolation and sometimes used to improve service quality (ie, the data are stored in a database for later reference during the genotyping process), whereas the consumers’ genome data are not used for inhouse research or sold to a third party for revenue. Moreover, it is often mandatory to store the sample (eg, for later reference of legal relationship tests). This cluster holds several representative service providers such as Alpha Biolabs, Dadchecksilver, or Who’z the daddy.

#### Cluster 4: Simple Health Tests

Cluster 4 combines chronic health-related genomic services such as Fulgent Diagnostics with the more casual health and wellness focused services such as SkinDNA Canada. These health-related services are either available through health professionals only or multicontact services, which may also include health professionals. This elaborate customer relationship leads to many services only operating locally, though some have a worldwide network. It comes as no surprise that this cluster contains most services aimed at chronic health issue and risk group consumers. Nevertheless, the cluster is still mainly populated with specific information seekers and enthusiast target groups. The 1-time fees are usually covered by the consumers but may be covered by insurances as well. The companies provide their own sampling kit and after genotyping, the sample is destroyed if storing it is not necessary. These services offer a lab collection option but come also with a home collection kit. Although the data ownership remains with the consumers and genome data are not sold for revenue or utilized in research, the results are mostly basic reports, which may require further interpretation by a local physician. Other examples of services in this cluster include International Bioscience or Pillcheck.

#### Cluster 5: Basic Low-Value Direct-to-Consumer Genomics

Cluster 5 differentiates itself from all other clusters in that it contains for profit as well as nonprofit companies that either charge a 1-time fee or offer services that are completely free. As services like Genetic Genie rely mostly on third-party sample collection, they can offer their services worldwide, exclusively via the internet as the consumers choose the sampling provider (eg, 23andMe) independently. The compatible sampling kits are for home collection only and aimed at genotyping tests. Consequently, the data ownership always lays with the consumers for services in this cluster and if stored, the genomic data are accessible for the costumers only. Furthermore, Genetic Genie does not sell the data for revenue, and there is no research consent option. As a result, the data processing is mostly done automatically, and the resulting interpretation is only of a basic nature. Nonetheless, enthusiasts may find a convenient way for additional insight into their genome. Other members of this cluster are Promethease, Roots for Real, or My Genetic Health.

#### Cluster 6: Comprehensive Tests and Low Data Processing

**Figure 4:**
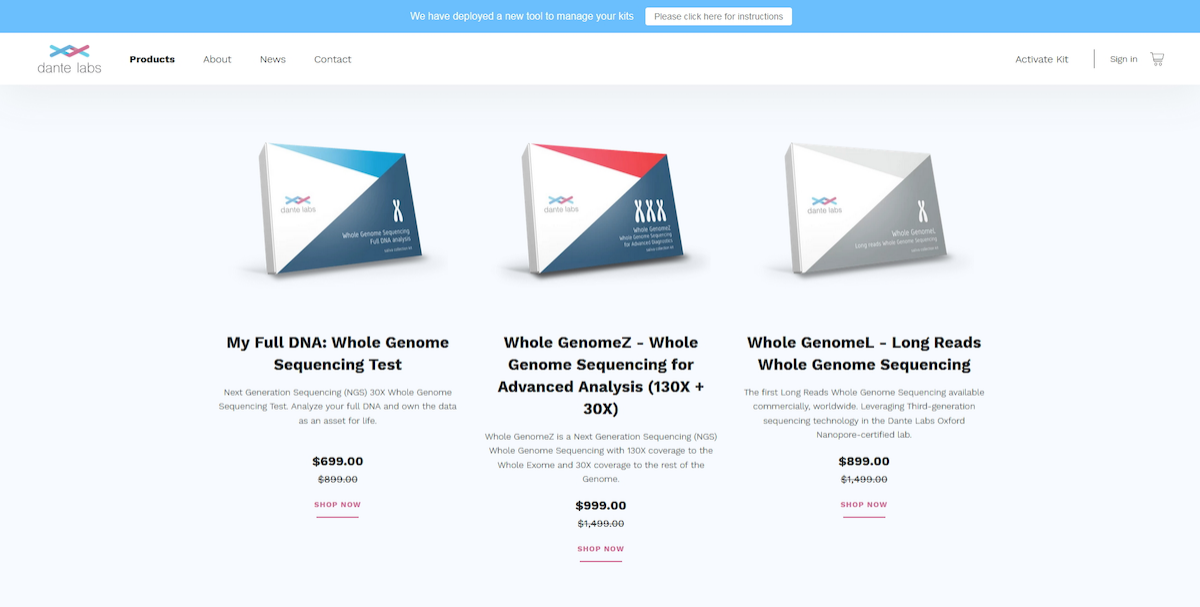
Screenshot of the Dante Labs website from October 2019.

Offering sequencing only or sequencing and genotyping tests, the last cluster holds services focusing on the sample processing mostly (ie, offering no or basic interpretation of the genome data only). Dante labs (see Figure 4 for a screenshot of their website), for example, offers a worldwide whole genome sequencing home collection kit via their website with a basic health analysis and Web access to the consumers’ entire sequenced genome. Other services in this cluster offer other forms of customer contact such as local labs or health professionals. The sampling kit may be provided by the service provider or additionally by a third party. Although this cluster has no specific target group, services are always paid for by the consumers with a 1-time fee. To compensate for the comprehensive genome tests and generate profit, companies often resell access to the produced genome data, make participation in research mandatory, or claim the right to use the data for company services. This is also mirrored in the data ownership, which may lie with the customer as well as with the company. Additional examples for services in this cluster are Full Genomes Corporation, Helix, or Genes for Good.

## Discussion

### Principal Findings

Analysis of the taxonomy and derived archetypes unveils interesting insights into the current state of the DTC genetic testing market.

First, our results paint a much more heterogeneous landscape of the DTC genetic testing market than most of the extant literature in this area has conceived. This is not only highlighted because of the presence of 6 diverse business model archetypes that we identified but also supported by the fact that the archetypes’ corresponding clusters are relatively evenly sized with cluster 5 being the smallest (n=21) and cluster 3 being the largest (n=73). Looking at the 5 business models described by Vanhala and Reijonsaari [29], the business model archetypes described here are rooted in a more diverse set of distinguishing dimensions (ie, 15 dimensions as opposed to 5 dimensions). This resulted in less (albeit still prevalent) emphasis on the different value propositions and consumer target groups and instead also included aspects such as major cost drivers (eg, dimensions such as sampling site and sampling kit provider). Furthermore, comparing the presented taxonomy with business model taxonomies of other disruptive technologies [30,48,49], we see that they share some similarities but also exhibit distinctive differences. Accordingly, our taxonomy includes several dimensions that are inherent to any business model, especially those dimensions that are related to customer segments, key partners, value propositions, or service pricing. Our fee-type dimension, for example, is comparable with the price structure dimension found in the carsharing business model taxonomy of Remane et al [30], albeit with slightly different characteristics. At the same time, however, the taxonomy presented here also includes several dimensions whose characteristics are more tailored toward the DTC genetic testing market (eg, distribution channel, fee payer), as well as dimensions that are entirely unique to the DTC genetic testing context (eg, genome test type).

Second, much of the controversy surrounding DTC genetic testing originates from concerns over the clinical value of such tests [50], consumers’ capabilities of dealing with potentially misleading test results [11], and the assumption that DTC genetic testing services sell access to their consumers’ genomic data to third parties [51]. To this end, cluster 1 seems to represent a business model archetype that many skeptics of DTC genetic testing services have in their minds when thinking of DTC genetic testing. This is further supported by the fact that the most prominent and probably most often criticized players in the DTC genetic testing market such as 23andMe, AncestryDNA, and FamilyTreeDNA can be found in this cluster. The business model archetype represented by cluster 1 also closely resembles the comprehensive genomic tests for consumers and as genome data bank material business model described by Vanhala and Reijonsaari [29]. Overall, however, our analysis showed that the majority of DTC genetic testing services do, for example, not resell access to their consumers’ genomic data to third parties for revenue (237 of 277). Even more so, cluster 2 represents a business model archetype where special emphasis is placed on consumers’ privacy. Several explanations might exist for these surprising, yet interesting, findings. Genomics, particularly DTC genetic testing, is still a relatively young business [28]. Next to the dimensions in the capture value category, the consumer research consent and data ownership dimensions are especially deeply related to a DTC genomics service provider’s profit as both dimensions exert a strong influence on what service providers can and cannot do with their primary resource, the produced genomic data. In this regard, it is important to note that scale benefits are mostly on the DTC genetic testing services’ side rather than the consumers’ side, as service providers can use already produced data to improve their service quality. Some service providers might also seek to incentivize interested individuals to use their service to establish a large enough database of genomic data that they can then use to develop complementary revenue streams, which do not directly involve selling access to genomic data to third parties (eg, use collected data to develop new drugs). Accordingly, some services might still be in a phase where growth is considered more important than short-term profit by the services’ stakeholders. Finally, some services might anticipate further declining costs for genome data collection and analysis because of technological advances, eventually making them more profitable and changing the relative benefits of different business models in the long run. An example for such a service is Veritas Genetics, who seek to provide whole genome sequencing services costing less than US $1000 to their consumers.

The third interesting finding pertains to recent debates about shifts in DTC genetic testing services’ strategies and the emergence of what some call DTC genomics 2.0. Compared with the currently prevailing DTC genomics paradigm, DTC genomics 2.0 is characterized by a greater involvement of regulatory bodies and health care professionals, a stronger separation between health and nonhealth tests, and improved support and counseling for consumers [6]. To this end, our clusters show a clear separation between services mainly targeting specific information seekers and people with chronic diseases (clusters 3 and 4), and those primarily targeting enthusiasts (clusters 1, 2, 5, and 6). Specifically, cluster 4 exhibits the largest number of services whose primary distribution channel are health care professionals and services whose tests are often paid for by consumers’ insurances. Cluster 4 represents a business model archetype that can serve as a prime example for a shift toward DTC genomics 2.0. Although such services arguably blur the lines between what is traditionally considered DTC genetic testing (ie, genetic tests, directly sold to consumers via the internet) and other forms of genetic testing (eg, in clinical or research settings), we think that the distinguishing factor is the primary recipient (ie, the consumers themselves and not clinicians) rather than the form of distribution or who actually pays for the tests. We also found several services (28 of 277) that offer genome sequencing to their consumers as opposed to only genotyping, potentially providing higher accuracy and clinical value than pure genotyping services. In consequence of these observations, our research supports the notion of an ongoing shift in the DTC genetic testing market to more mature DTC genomics 2.0.

Fourth, although most DTC genetic testing services were profit oriented, we found it interesting that there were at least 14 nonprofit services in our sample. Although these services did not form their own cluster in our 6-cluster solution, the majority of these services (n=9) can be found in cluster 5. They almost exclusively operate worldwide, only over the internet, and only offer third-party home collection kits and genotyping services. A notable exception to this is the DTC genetic testing service of Genes for Good that operates only in the United States, provides its own home collection kit and offers sequencing services. It is a nonprofit organization run by the University of Michigan, which seeks to engage people in genetic research. Generally, nonprofit DTC genetic testing services heavily rely on other for-profit DTC genetic testing services by, for example, using the test kits of other services or directly requesting consumers to import their data from other services. Specifically, the last scenario, where consumers freely and openly share their genome data with for-profit and nonprofit services, has received attention from researchers interested in socioeconomic perspectives of genome data sharing and crowdsourcing [1,24] and could be seen as an indicator for a trend toward *platformization* and a platform economy in genomics.

### Limitations

Limitations of our study are as follows. First, the DTC genetic testing market is a volatile market with new services regularly appearing, mergers and acquisitions constantly happening, and extant services disappearing or changing their business strategies. Our taxonomy and archetypes, however, represent a snapshot of the current landscape of DTC genetic testing service business models. It is likely that in the meantime, new services would have emerged, while some services in our sample would have changed their business models or completely disappeared from the market. Nevertheless, we are confident that the developed taxonomy and derived archetypes build a strong foundation for further research in this area because of the rich and meaningful sample of DTC genetic testing services used and rigorous development of both. Moreover, owing to the extendibility ending condition being met, the presented taxonomy can easily be extended or altered in the case of the emergence of new services or additional insights. Second, our examination of services was mainly based on information retrieved from nonscientific sources and services’ internet presences. Some information provided was ambiguous or not given at all. It is possible that our coding of some of the services are not entirely appropriate. We sought to address these information deficits by having services examined by 2 researchers independently, consulting additional internet sources, and, where no information was found, making informed guesses by comparing specific services with other similar services. Third, although Ward’s method indicated the validity of several cluster solutions, analysis of common metrics provided inconclusive results on which cluster solution to choose for the *k*-means algorithm (ie, the dendrogram suggested 4, 5, and 6 cluster solutions, whereas the scree plot suggested 4, 5, 7, and 9 cluster solutions). After thorough examination of these cluster solutions based on a *k*-means clustering, we deemed the 6-cluster solution to be the most promising one for this research. Nevertheless, the 7-cluster solution might have provided additional insights into the DTC genomics market, which are not captured by the current cluster solution.

### Implications and Future Research

Our study yields several implications for research and practice. For research, we provide a systematic classification of DTC genetic testing service business models and expose their essential characteristics. The developed taxonomy adds to our knowledge of business models in genomics, and in conjunction with the proposed archetypes, it contributes to a more comprehensive understanding of the DTC genetic testing industry. Compared with other taxonomies about technologies that are similarly disruptive as DTC genetic testing [35], the taxonomy presented here focuses on a rather narrow aspect of the DTC genetic testing service phenomenon. Although the objective of this research was specifically to analyze an area of DTC genetic testing that has received little attention from researchers thus far (ie, the business models in this very industry), we also think that the presented taxonomy can serve as a starting point to analyze the DTC genetic testing phenomenon as a whole by, for example, broadening the taxonomy’s scope and further developing it into a general taxonomy of DTC genetic testing services. However, we also note that despite the seminal work of Hwang and Christensen [8], the literature on the classification of business models in health care remains scarce. The presented taxonomy and archetypes can serve as an outset for new avenues in future research on DTC genetic testing. Starting from the previously outlined limitations of this study, researchers could seek to replicate the results of our research, especially considering the likely emergence of new DTC genetic services. Furthermore, as the taxonomy and archetypes only capture a snapshot of current DTC genetic testing service business models, future research should also attempt to analyze how those services’ business models change over time. To this end, the literature on business model innovation [52,53] could provide a promising foundation to analyze the evolution of business models in DTC genetic testing. Although the clusters presented in this work were developed independent of any temporal dimension, future research may also investigate whether these clusters relate to different evolutionary stages of DTC genetic testing business models. From a socioeconomic and genetic privacy research perspective, our research provides a starting point to better understand the economic value of genomic data. We thereby support and strengthen the notion of information privacy as a commodity that can be traded in the context of genomics [54]. Specifically, dimensions in the categories such as strategic choices, create value, and capture value might prove useful to better understand the relationships between business models, genetic privacy, and crowdsourcing in genomics.

In terms of practice, our research has important implications for policy makers, professionals in the health care industry, DTC genetic testing services themselves, and consumers of such services. For policy makers, our research highlights a reality of diverse business models within the DTC genetic testing market. Considering DTC genetic testing services’ need to collaborate closely with regulatory bodies, the developed taxonomy and archetypes can assist policy makers in designing policies that adapt to the diverse business landscape as to better protect consumers’ well-being and privacy while respecting their right to informational self-determination. Similarly, the archetypes of DTC genetic testing service business models help raise awareness for the existence of different kinds of services with diverse benefits and risks for consumers and the health care system. Overall, our work contributes to a more nuanced understanding of DTC genetic testing in the health care sector, which has become progressively important given that evidence from studies among U.S. populations (one of the largest DTC genetic testing markets) suggests patients increasingly talk to their primary care physicians about DTC genetic testing [55,56], and expect them to be able to answer questions about DTC genetic test results [57]. For DTC genetic testing services, especially young services, our taxonomy serves as a valuable tool to analyze and possibly further develop their own business models as well as to analyze competitors’ business models. The presented archetypes provide decision makers of DTC genetic testing services with blueprints of potential business models. Such blueprints can be used as initial guidance for transitioning from 1 business model to another or to identify market niches. From a consumer’s point of view, it was a pleasant surprise that most DTC genetic testing service providers do not resell access to their consumers’ genomic data to third parties. However, at the same time, our analysis reveals that many of the examined DTC genetic testing services retain the right to use collected genomic data for service improvements, something which consumers should be aware of. Overall and in line with research seeking to understand and improve consumers’ understanding of DTC genetic testing [13,14], the taxonomy and archetypes presented in this study can serve consumers as tools for assessing DTC genetic testing services and finding services that best fit their needs.

### Conclusions

DTC genetic testing is a relatively young and dynamic business area, which pushes genomics research forward and promises faster, as well as more affordable genomics services. Despite the rapid growth of the business sector, many concerns remain unanswered, and there is little knowledge about the impact of business models on DTC genetic testing services within research literature. In this study, we provide the first overview of DTC genetic testing service business models, resulting in a rigorously developed taxonomy and 6 service archetypes. This provides novel insights into the value and use of genomic data and can serve as a foundation for advanced research on relationships among DTC genetic testing services, their consumers, practitioners within the health care sector, and policy makers.

## Appendix

Multimedia Appendix 1 Overview of included and excluded direct-to-consumer genetic testing services.

Multimedia Appendix 2 Taxonomy development approach.

Multimedia Appendix 3 Taxonomy development iterations details.

Multimedia Appendix 4 Cluster analysis details.

Multimedia Appendix 5 Taxonomy coding overview.

Multimedia Appendix 6 Cluster analysis details.

## Abbreviations

DTC: direct-to-consumer
FDA: US Food and Drug Administration
