# Supplementary material for: Valuable Genomes: Taxonomy and Archetypes of Business Models in Direct-to-Consumer Genetic Testing
Source: J Med Internet Res. 2020 Jan 21;22(1):e14890. doi: 10.2196/14890 (PMC7001042; doi:10.2196/14890)
Supplement: Multimedia Appendix 2 [file jmir_v22i1e14890_app2.pdf]

Multimedia Appendix 2. Taxonomy development approach.

We based the development of our taxonomy on the method by Nickerson et al [1]. It consists of seven iterative steps and provides guidelines for each step in the taxonomy development. Figure MA2-1 gives an overview of the seven steps, while a detailed description of each step is given below.

Figure MA2-1: Taxonomy development approach by Nickerson et al [1].

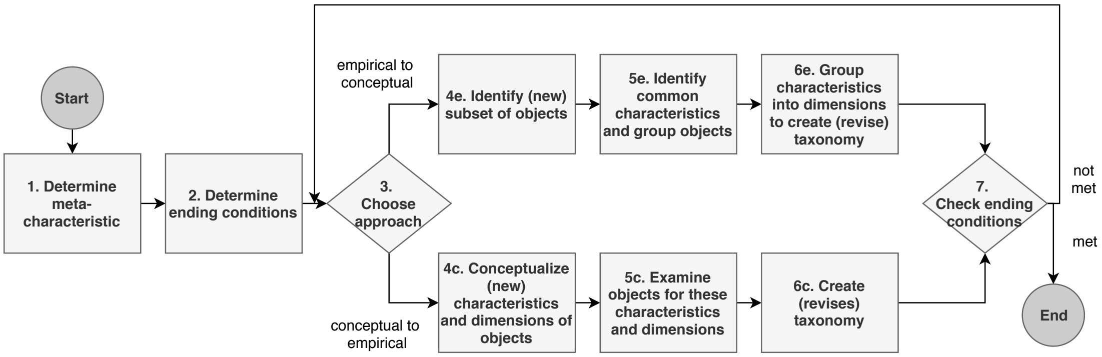

Overview

Step 1: Meta-Characteristic

The first step of the method by Nickerson et al [1] abides the selection of a so-called meta-characteristic for the taxonomy development process. Determining characteristics of the objects of interest is an integral part of the taxonomy development. However, researchers might tend to examine a large number of related and unrelated characteristics in hopes of discovering a pattern. The meta-characteristic serves to avoid this situation of naïve empiricism and provides a basis for identifying dimensions and characteristics relevant to the taxonomy. Being the most comprehensive characteristic, the meta-characteristic acts as the basis for the choice of characteristics in the taxonomy. Thus, each characteristic must be a logical consequence of the meta-characteristic. For the development of this taxonomy the meta-characteristic is defined as *salient properties of business models of DTC genetic testing services*.

Step 2: Ending Conditions

Since steps three to seven of the taxonomy development approach are of an iterative nature, some predetermined conditions that end the iterative process have to be defined. For this research, we adopt the eight objective ending conditions and five subjective ending conditions provided by Nickerson et al [1], which are as follows.

Objective ending conditions:

1. Conciseness: A taxonomy should comprise a limited number of dimensions and characteristics in each dimension in order to ensure its utility and ease of use.

2. Robustness: A taxonomy should contain a sufficient number of dimensions and characteristics that allow for adequately differentiating objects of interest from each other.
3. Comprehensiveness: A conceptually developed taxonomy should contain all relevant dimensions for the objects of interest. An empirically developed taxonomy should allow for classifications of all objects of interest.
4. Extendability: It must be possible to adapt a taxonomy to the continuous changes in a domain (ie, by adding, removing, or changing dimensions).
5. Explanatory: The taxonomy should be explanatory in a sense that it serves to aid in understanding related objects in a complex area of interest.

Subjective ending conditions:

1. All objects of interest or a representative sample thereof have been examined.
2. No object was merged with a similar object or split into multiple objects in the last iteration. Since, a merge or split might affect changes to the objects, a review of the corresponding dimensions and characteristics is necessary.
3. At least one object is classified under every characteristic of every dimension. If a characteristic without a matching object exists, there is a so-called 'null' characteristic in the taxonomy, which needs to be removed.
4. No new dimensions or characteristics were added in the last iteration. If new dimensions or characteristics were added, it may be possible to find additional dimensions or characteristics in the following iteration.
5. No dimensions or characteristics were merged or split in the last iteration. A merge or split might affect changes to dimensions and characteristics, which require a review of the other dimensions and characteristics.
6. Every dimension is unique. Redundancies among dimensions need to be removed to maintain the uniqueness of the taxonomy.
7. Every characteristic is unique within its dimension. Redundancies among characteristics in the same dimension need to be removed to maintain the uniqueness of the taxonomy.
8. Each cell (ie, combination of characteristics) is unique. Redundant cells in the taxonomy need to be removed to maintain the uniqueness of the taxonomy.

Steps 3-7: Iteration

After the subjective and objective conditions have been determined in step 2, the first iteration starts by choosing either a conceptual-to-empirical (deductive) or empirical-to-conceptual (inductive) approach in step 3. For the conceptual-to-empirical approach the researcher conceptualizes the dimensions and characteristics of the taxonomy without examining actual objects. This fourth step (step 4c) is based on the researchers own knowledge and/or the accomplishments of previous studies. Thereafter, the identified dimensions and characteristics must be validated with objects of interest in step 5c. All validated dimensions and characteristics can then be formed to build an initial taxonomy or added to an existing taxonomy for step 6c. The empirical-to-conceptual approach in contrast starts by identifying a subset of objects of interest in step 4e. This can either be a random sample, systematic sample, a convenience sample, or some other type of sample. Next, the researchers identify

common characteristics from the sample of objects and group the objects accordingly in step 5e. Forming these groups into dimensions with characteristics they can either be used to build the initial taxonomy or merged with an existing taxonomy from previous iterations in step 6e. Regardless of the chosen approach in step 3, each iteration must be completed by reviewing whether the ending conditions from step 2 were met in step 7. If they were not met another iteration starting from step 3 is necessary. In total, we per- formed six iterations. An overview of the taxonomy development iterations performed for this research (including the chosen approach) can be found in Multimedia Appendix 3

References

1. Nickerson RC, Varshney U, Muntermann J. A method for taxonomy development and its application in information systems. European Journal of Information Systems. 2013;22(3):336-59.
