# Supplementary material for: Valuable Genomes: Taxonomy and Archetypes of Business Models in Direct-to-Consumer Genetic Testing
Source: J Med Internet Res. 2020 Jan 21;22(1):e14890. doi: 10.2196/14890 (PMC7001042; doi:10.2196/14890)
Supplement: Multimedia Appendix 3 [file jmir_v22i1e14890_app3.pdf]

Multimedia Appendix 3. Taxonomy development iterations details.

Table MA3-1: Overview of individual taxonomy development iterations.

Iter.	Approach	Source(s) / Objects	Dimension	Rationale	Ending cond.
1	C2E	Vanhala and Reijonsaari [1]	Distribution Channel	Basic dimensions acquired by reviewing the classification of Vanhala and Reijonsaari [1] and adopting or refining their dimensions	Not met
			Fee type		
			Fee payer		
			Revenue from genome data		
			Consumer target group		
			Genome test type		
			Data ownership		
			Data processing		
2	E2C	Top 10 Ancestry, Paternity, Health from DNA Testing Choice	Consumer research consent	Some inspected services offer voluntary sharing of personal genome data for research, while for other services such sharing is mandatory	Not met
			Data storage	Privacy policies state how genome data is stored or who has access	
3	E2C	Tests in genomics enthusiasts category	Business purpose	Inspection of non-profit services required a distinction to for-profit services	Not met
			Region of operation	Some services, although online accessible worldwide will only sell tests in their respective country	
			Sample storage	Terms of service state if and for how long the genome sample is stored or accessed	
4	C2E	-	Genome sample	Different methods of sampling (eg. blood, buccal swab, saliva sample) which require different collection sites (eg, home, lab, mobile collection) were discovered in previous iterations	Not met
5	E2C	Tests in relationship category	Legal option	Relationship test may have special legal requirements	Not met
6	C2E	-	Sampling site	Genome sample dimension was not mutually exclusive and thus split into two dimensions	Not met
			Sampling kit provider		
			Data processing	The Legal option dimension needed to be aligned with and was therefore integrated into the Data processing dimension	
7	E2C	Tests in health category	No changes to the taxonomy, just new objects classified	-	Met

E2C: Empirical-to-conceptual, C2E: Conceptual-to-empirical

References

1. Vanhala A, Reijonsaari K. Direct-to-consumer genome data services and their business models. Sitra; 2013 [2018/04/20]; Available from: https://media.sitra.fi/2017/02/28142338/Direct_to_consumer_genome_data_services_and_their_business_models.pdf archived at: http://www.webcitation.org/78NrCMxLB.
