# Supplementary material for: Valuable Genomes: Taxonomy and Archetypes of Business Models in Direct-to-Consumer Genetic Testing
Source: J Med Internet Res. 2020 Jan 21;22(1):e14890. doi: 10.2196/14890 (PMC7001042; doi:10.2196/14890)
Supplement: Multimedia Appendix 4 [file jmir_v22i1e14890_app4.pdf]

Multimedia Appendix 4. Cluster analysis details.

Table MA4-1: k-means evaluation table.

		Cluster solution	4	5	6	7	9
		Number of iterations[a]	10	17	9	13	12
		Theoretical Ø Cluster Size[b]	69	55	46	39	30
		Small Clusters' Size[c]	28	27	21	12;15	11;12;14
		Number of clusters supported by dendrogram	Yes	Yes	Yes	No	No
		Number of clusters supported by elbow rule	Yes	Yes	No	Yes	Yes
		Number of characteristics with *Sig.* ≥ 0.001[d]	11	12	4	6	4
Cat.	Dimension	Characteristic	*Sig.*				
Strategic Choices	Business purpose	For profit	.001	.000	.000	.000	.000
		Non-profit	.001	.000	.000	.000	.000
	Region of operation	Local	.000	.000	.000	.000	.000
		Worldwide	.000	.000	.000	.000	.000
	Consumer target group	Enthusiasts	.000	.000	.000	.000	.000
		Specific information seekers	.000	.000	.000	.000	.000
		Enthusiasts and specific information seekers	.094	.035	.578	.003	.350
		Chronic health issue and risk group	.000	.007	.000	.000	.000
	Consumer research consent	Mandatory	.000	.000	.000	.000	.000
		Optional	.000	.000	.000	.000	.000
		Data not used	.000	.000	.000	.000	.000
Value Network	Distribution channel	Internet only	.000	.000	.000	.000	.000
		Health care professionals only	.000	.007	.000	.000	.000
		Multi-contact service	.000	.000	.000	.000	.000
	Sampling site	Home collection	.000	.000	.000	.000	.000
		Lab collection	.000	.004	.000	.000	.000
		Home and lab collection	.000	.000	.000	.000	.000
	Sampling kit provider	Service provider	.000	.000	.000	.000	.000
		Third party	.000	.000	.000	.000	.000
		Service provider and third party	.022	.016	.045	.000	.057
	Sample storage	Never	.000	.000	.000	.000	.000
		Mandatory	.000	.000	.000	.000	.000
		Consumer decision	.000	.000	.000	.000	.004
Create Value	Genome test type	Genotyping	.000	.001	.000	.000	.000
		Sequencing	.173	.043	.069	.379	.146
		Genotyping and sequencing	.001	.010	.000	.001	.000
	Data storage	No storage	.000	.000	.000	.000	.000
		Isolated storage	.000	.000	.000	.000	.000
		Database for company services	.000	.000	.000	.000	.000
	Data ownership	Consumer	.000	.000	.000	.000	.000
		Service provider	.000	.000	.000	.000	.000
	Data processing	No interpretation	.139	.203	.004	.329	.000
		Basic interpretation	.006	.000	.000	.000	.000
		Value added interpretation	.008	.000	.000	.000	.000

		Cluster solution	4	5	6	7	9
Cat.	Dimension	Characteristic	*Sig.*				
Capture Value	Fee type	Pay-per-use	.000	.000	.000	.000	.000
		Pay-per-use and subscription	.001	.023	.000	.008	.000
		No fee	.004	.000	.000	.003	.000
	Fee payer	Consumer only	.000	.001	.000	.000	.000
		Consumer and health insurance	.000	.001	.000	.000	.000
	Reselling of genome data	Yes	.000	.000	.000	.000	.000
		No	.000	.000	.000	.000	.000

a. Fewer iterations indicate more stable cluster partitions, as convergence is achieved quicker.
b. Calculated by dividing n = 277 objects by the number of desired clusters k. This is the theoretical average size of each cluster.
c. Only showing clusters that are below 50% of the Theoretical Ø Cluster Size or the single smallest cluster size. Small clusters have less explanatory power/not enough objects to deduct meaningful archetypes. Nonetheless, a small cluster might just be underrepresented, and a larger sample size could allow meaningful interpretation, if the cluster increases.
d. ANOVA results show significance values (*Sig.*) for each variable (ie, characteristic of the taxonomy) with 0 ≤ Sig. ≤ 1. A low *Sig.* indicates that the characteristic is relevant for the cluster solution. Thus, the optimal cluster solution should have few Sig. > 0.
