# Supplementary material for: Valuable Genomes: Taxonomy and Archetypes of Business Models in Direct-to-Consumer Genetic Testing
Source: J Med Internet Res. 2020 Jan 21;22(1):e14890. doi: 10.2196/14890 (PMC7001042; doi:10.2196/14890)
Supplement: Multimedia Appendix 6 [file jmir_v22i1e14890_app6.pdf]

Multimedia Appendix 6. Cluster analysis details.

Table MA6-1: Crosstab analysis results.

Dimensions[a]	Characteristics[b]	Cluster 1	Cluster 2	Cluster 3	Cluster 4	Cluster 5	Cluster 6
Business purpose	For profit (%)	91	100	100	100	57	96
	Nonprofit (%)	9	0	0	0	43	4
Region of operation	Worldwide (%)	89	83	52	29	100	75
	Local (%)	11	17	48	71	0	25
Consumer target group	Enthusiasts (%)	79	78	0	17	76	64
	Specific information seekers (%)	6	8	84	44	10	14
	Enthusiasts and specific information seekers (%)	6	7	15	10	10	7
	Chronic health issue and risk group (%)	9	7	1	29	5	14
Consumer research consent	Mandatory (%)	34	2	5	6	0	29
	Optional (%)	47	12	5	4	5	64
	Data not used (%)	19	87	89	90	95	7
Distribution channel	Internet only (%)	70	57	4	6	100	82
	Health care professionals (%)	6	8	0	29	0	7
	Multi-contact service (%)	23	35	96	65	0	11
Sampling site	Home collection (%)	87	82	8	46	100	79
	Lab collection (%)	0	2	10	25	0	4
	Home and lab collection (%)	13	17	82	29	0	18
Sampling kit provider	Service provider (%)	57	80	96	92	19	89
	Third party (%)	21	10	1	0	71	0
	Service provider and third party (%)	21	10	3	8	10	11
Sample storage	Never (%)	51	65	4	81	48	46
	Mandatory (%)	15	18	88	10	5	32
	Consumer decision (%)	34	17	8	8	48	21
Genome test type	Genotyping (%)	87	95	100	92	90	54
	Sequencing (%)	2	2	0	2	5	11
	Genotyping and sequencing (%)	11	3	0	6	5	36
Data storage	No storage (%)	0	37	63	65	48	4
	Isolated storage (%)	0	53	14	10	43	18
	Database for company services (%)	100	10	23	25	10	79
Data ownership	Consumer (%)	66	100	93	96	100	71
	Company (%)	34	0	7	4	0	29
Data processing	No interpretation (%)	0	3	0	0	0	11
	Basic interpretation (%)	0	3	7	73	100	86
	Value added interpretation (%)	100	93	93	27	0	4
Fee type	No fee (%)	4	0	0	0	33	4
	Pay per use (%)	77	88	100	100	62	96
	Pay per use & subscription (%)	19	12	0	0	5	0
Fee payer	Consumer only (%)	89	100	99	77	100	96
	Consumer and health insurance (%)	11	0	1	23	0	4
Reselling of genome data	Yes (%)	47	0	8	0	5	39
	No (%)	53	100	92	100	95	61
	**Cluster Size**	47	60	73	48	21	28

a. All characteristics of one dimension sum up to 100% as characteristics are mutually exclusive and collectively exhaustive [1].
b. The percentage of a characteristic for a cluster is the number of applicable items divided by the size of the cluster.

Darker colors represent higher percentages
0% 100%

References

1. Nickerson RC, Varshney U, Muntermann J. A method for taxonomy development and its application in information systems. European Journal of Information Systems. 2013;22(3):336-59.
